# Simple yet (more?) effective. Venous thromboembolism risk assessment model for germ cell tumour patients receiving first‐line chemotherapy

**DOI:** 10.1002/cam4.6458

**Published:** 2023-08-16

**Authors:** Wojciech Michalski, Grażyna Poniatowska, Joanna Jońska‐Gmyrek, Agnieszka Żółciak‐Siwińska, Inga Zastawna, Artur Lemiński, Anna Macios, Michał Jakubczyk, Tomasz Demkow, Paweł Wiechno

**Affiliations:** ^1^ Department of Urological Cancer Maria Sklodowska‐Curie National Research Institute of Oncology Warsaw Poland; ^2^ Department of Gynaecological Oncology Maria Sklodowska‐Curie National Research Institute of Oncology Warsaw Poland; ^3^ Clinical Centre of Cardiology and Rare Diseases of the Cardiovascular System National Institute of Medicine of the Ministry of the Interior and Administration Warsaw Poland; ^4^ Department of Urology and Urological Oncology Pomeranian Medical University Szczecin Poland; ^5^ Department of Cancer Prevention Maria Sklodowska‐Curie National Research Institute of Oncology Warsaw Poland; ^6^ SGH Warsaw School of Economics, Institute of Econometrics, Collegium of Economic Analysis Warsaw Poland

**Keywords:** deep vein thrombosis, germ cell cancer, pulmonary embolism, risk factors

## Abstract

**Background:**

Germ cell tumours (GCT) are highly curable malignancies. Venous thromboembolism (VTE) is a serious complication, needing better risk assessment models (RAM).

**Aim:**

Identification of VTE incidence and risk factors in metastatic GCT patients starting first‐line chemotherapy. Developing a RAM and comparing it to Khorana risk score (KRS) and Padua Prediction Score (PPS).

**Material and methods:**

We retrospectively analysed GCT patients staged IS–IIIC. VTE risk factors were identified with logistic regression. Area under curve of receiver operating characteristic (AUC‐ROC), Akaike and Bayesian Information Criteria (AIC, BIC) were calculated for the developed RAM, KRS and PPS.

**Results:**

Among 495 eligible patients, VTE occurred in 69 (13.9%), including 40 prior to chemotherapy. Vein compression (OR: 8.96; 95% CI: 2.85–28.13; *p* < 0.001), clinical stage IIIB‐IIIC (OR: 5.68; 95% CI: 1.82–17.70; *p* = 0.003) and haemoglobin concentration (OR for 1 g/dL decrease: 1.32; 95% CI: 1.03–1.67; *p* = 0.026) were significant in our RAM. KRS ≥ 3 (OR: 3.31; 95% CI: 1.77–6.20; *p* < 0.001), PPS 4–5 (OR: 3.06; 95% CI: 1.49–6.29; *p* = 0.002) and PPS > 5 (OR 8.05; 95% CI 3.79–17.13; *p* < 0.001) correlated with VTE risk. Diagnostic criteria (AUC‐ROC, AIC, BIC) for the developed RAM, KRS and PPS were (0.885; 0.567; −1641), (0.588; 0.839; −1576) and (0.700; 0.799; −1585), respectively. In the numerical score, the optimal cut‐off point for high‐risk was ≥9, with sensitivity, specificity, positive and negative predictive value of 0.78, 0.77, 0.35 and 0.96, respectively.

**Conclusions:**

Our RAM, based on vein compression, clinical stage and haemoglobin concentration proved superior to both KRS and PPS. VTE is frequent in GCT patients.

## Abstract 2

We identified venous thromboembolism (VTE) risk factors in a group of 495 germ cell tumour patients staged IS–IIIC and constructed prediction models. In the best model, vein compression, stage IIIB–IIIC and lower haemoglobin concentration predicted VTE risk. Our risk assessment model proved superior to Khorana risk score and Padua Prediction Score; a numerical risk score is to be further validated.

## 1 BACKGROUND

Germ cell tumours (GCT) account for the majority of malignancies in men aged 15–44.^1^ They mostly originate from testicles (testicular germ cell tumours, TGCT) and, in 5% cases, from extragonadal tissues localised in the body's midline, that is, retroperitoneal space, mediastinum or central nervous system (CNS).^2^ According to Znaor et al,^1^ an increase in GCT incidence is prognosed in 20 European countries. Until 2035, a total number of GCT patients is bound to increase by 21%, 13% and 32% in Northern, Western and Eastern Europe, respectively. A decrease by 12% is prognosed for Southern Europe.^1^

Curability of GCT is high; it amounts to 100% in clinical stage (CS) I (disease limited to the testis). Multiagent chemotherapy is also curative in disseminated cases so that 80% of patients achieve a durable remission.^2^ To maintain these outcomes, referring patients to centres of excellence is recommended, particularly advanced or recurrent cases.^1^

Due to the good prognosis and patients' young age, there has been a shift towards reducing treatment burden, as well as cancer‐unrelated morbidity and mortality. Venous thromboembolism (VTE), comprising deep vein thrombosis (DVT) and pulmonary embolism (PE), has been a marked villain there.^3^ Nuances in pathophysiology (e.g. involvement of cancer cells, pro‐coagulant state, oncological treatment) and clinical issues (e.g. frequent thrombocytopenia, bleeding, central venous access devices) support the term cancer‐associated thromboembolism (CAT).^3^, ^4^ VTE may be the first symptom of cancer^5^, ^6^; 4%–20% cancer patients are predicted to experience VTE at some stage of their disease. VTE events within the first year from diagnosis confer poorer prognosis.^4^ A broad spectrum of clinical presentations, from asymptomatic cases or incidental computed tomography (CT) findings to sudden cardiac arrest,^7^, ^8^ makes VTE diagnosis challenging. Therefore, VTE risk assessment and thromboprophylaxis are crucial, especially in curable malignancies. Khorana risk score (KRS)^9^ and Padua Prediction Score (PPS)^10^ are among the most recognised risk assessment models (RAM). However, KRS identifies high‐risk patients better and is still suboptimal in low‐ and intermediate‐risk groups. Meanwhile, it is in the latter that majority of VTE events occur.^11^, ^12^

The established RAMs will remain the benchmark for future studies in this field. However, we feel that a more precise CAT prediction should rely on newer scores, specific for different primary sites and histopathology.

## 2 AIM

The aim of this study was to determine VTE incidence and clinical presentations among GCT patients commencing first‐line chemotherapy, and to identify potential histological, clinical and laboratory risk factors. We also aimed to assess the performance of KRS and PPS in this cohort, and, finally, construct a RAM specifically dedicated to GCT patients.

## 3 MATERIAL AND METHODS

### 3.1 Patients' selection

We retrospectively searched our clinical databases for male patients with gonadal and extragonadal (retroperitoneal, mediastinal and intracranial) GCT (International Classification of Diseases, ICD‐10 codes: C62, C48, C38 and C71, respectively) who had undergone first‐line chemotherapy at our institution between 2011 and 2019. Patients with disseminated disease, that is, CS IS–IIIC according to TNM (eighth edition), who commenced multiagent chemotherapy or at least prephase (if applied) were eligible. Of note, TNM classifications for primary retroperitoneal (C48) and mediastinal (C38) tumours are designed mainly for soft tissue sarcomas. Moreover, tumours of the central nervous system (C71) are not routinely staged with TNM system; their dissemination is defined as radiological lesions along the cerebrospinal axis or positive cerebrospinal fluid cytology. For the sake of this study, we adapted TNM used for TGCT (C62) to describe extragonadal GCT as well (i.e. T0 N0‐3 M0‐1 S0‐3) to maintain consistent staging.

The study was approved by the Ethical Committee at Maria Sklodowska‐Curie National Research Institute of Oncology, Warsaw, Poland (consent no. 16/2021; 25 March, 2021).

### 3.2 Material

The following data were extracted:

- Demographics: age, body mass, height, body surface area (BSA), body mass index (BMI), Eastern Collaborative Oncology Group (ECOG) performance status, history of VTE, thrombophilia and smoking.
- Disease‐related: clinical stage, International Germ Cell Cancer Collaborative Group (IGCCCG) prognosis, histological components, localisation of the primary focus and metastases, the biggest dimension of retroperitoneal and mediastinal lesions.
- Treatment‐related: adjuvant chemotherapy and/or radiotherapy for stage I disease (if applicable), prior surgery (orchiectomy, laparotomy, mediastinotomy, craniotomy or other), first‐line chemotherapy (regimen, number of cycles), central venous access, extended (between cycles) VTE prophylaxis, granulocyte colony‐stimulating factor (G‐CSF) administration.
- Laboratory: haemoglobin (Hgb), platelet (Plt) count, mean platelet volume (MPV), leukocyte (WBC), neutrocyte (Neutr), lymphocyte (Lym) and monocyte (Mono) counts, platelets‐lymphocytes ratio (PLR), neutrocytes–lymphocytes ratio (NLR), glomerular filtration rate (GFR), fibrinogen, activated partial thromboplastin time (aPTT), D‐dimer, serum tumour markers: alpha‐foetoprotein (AFP), human chorionic gonadotropin (hCG) and lactate dehydrogenase (LDH);
- KRS and PPS.
- Endpoint: overt DVT and/or PE; time (days) from chemotherapy start to the event.

Due to the retrospective nature of this study, VTE was reported:

- On CT, performed routinely for staging or follow‐up purposes (in asymptomatic cases).
- On Doppler ultrasound, performed when DVT was suspected clinically.
- On CT angiography, performed when PE was suspected clinically.
- In medical records from the follow‐up period.

The medical records were screened for VTE up to 6 months after the end of first‐line chemotherapy or until residual lesions surgery, second‐line chemotherapy, any radiotherapy or patient's death, whichever occurred first. Variables were coded as continuous (age, BSA, BMI, dimensions of retroperitoneal and mediastinal lesions; all laboratory parameters), ordinal (IGCCCG risk group, PPS: >5 and 4–5 vs. 3) or binary (CS: IIIB–IIIC vs. IS–IIIA, KRS: 3–5 vs. 1–2, and all the remaining).

### 3.3 Statistics

Stata®15.1 software was used. Potential VTE risk factors were compared between two subgroups, that is, patients with and without VTE. The final set of patients was determined by a dynamic selection of variables, including these with missing data. Significant variables were indicated by the algorithm, which then excluded patients with missing data in these very variables.

VTE risk factors were identified by logistic regression, with significance threshold of 0.05. Variables were first subjected to univariate analyses.

The number of variables subjected to multivariate analyses was limited by the number of VTE events (one variable per 10 events). They were chosen on the basis of univariate analyses and subject‐matter‐knowledge approach. We analysed six models of seven variables each, with *p* < 0.05 as significance threshold. All the ‘complete models’ were subjected to forward stepwise regression, with 0.3 as significance threshold, to form three new ‘selected models’. The best ‘complete’ and ‘selected’ model was compared to KRS and PPS in terms of area under curve of receiver operating characteristics (AUC‐ROC) statistics as well as Akaike and Bayesian information criteria (AIC and BIC). We applied the following interpretation of AUC‐ROCs: 0.5 – random, 0.5–0.7 – low, 0.7–0.8 – acceptable, 0.8–0.9 – good/very good, >0.9 – excellent predictive value.^13^

Subsequent analyses were conducted post hoc, after the best‐fitting model was found. It comprised Hgb as a continuous variable. We aimed to investigate whether converting Hgb into a binary variable would yield better VTE prediction. Hgb values were arranged according to quartiles. This enabled to assess the coefficient of Hgb as the only explanatory variable in each quartile. Moreover, Hgb concentration was subjected to locally weighted scatterplot smoothing analysis (LOWESS). Estimates of binarily coded Hgb were then introduced into multivariate models and compared to the original model with Hgb as a continuous variable, with odds ratios (OR) calculated for 1 g/dL decrease.

Finally, we assigned weights (according to regression coefficients and odds ratios in multivariate analyses) to the items of the proposed RAM. A cut‐off point for low/high VTE risk was estimated and remains to be confirmed in a validation cohort.

Ordinal variables were compared with chi‐squared test; continuous variables were first checked for normality of distribution with Shapiro–Wilk test. Further comparisons were conducted with Student's *t* test or Mann–Whitney–Wilcoxon test, for variables with normal or non‐normal distributions, respectively.

Continuous variables were presented as means and standard deviations; numerical variables—as numbers and percentages.

## 4 RESULTS

### 4.1 Patients' characteristics

We identified 504 patients fulfilling the inclusion criteria, nine of whom were lost to follow‐up after treatment; 495 patients were hence eligible for analyses. In 17 (3.4%) patients, treatment was commenced on the basis of clinical presentation and unequivocal markers concentrations indicating GCTs. Extended thromboprophylaxis with a low molecular weight heparin (LMWH) was administered in 173 (34.9%) patients. There were missing data in several variables. Smoking was assessed in 308 (62.2%) patients, histopathology in 478 (96.6%), burned‐out tumour (pathologically or radiologically) in 492 (99.4%), biggest diameter of retroperitoneal lesion in 490 (99.0%), biggest dimension of mediastinal lesion in 490 (99.0%), vessels compression – 369 (74.5%), D‐dimer concentration – 426 (86.1%), aPTT – 413 (83.4%), fibrinogen – 351 (70.9%), AFP – 492 (99.4%), hCG – 492 (99.4%) and LDH – in 494 (99.8%) patients. Detailed data are presented in Tables 1 and 2.

**TABLE 1:** Patients' characteristics (percentages of subgroups with complete data).

Variable	VTE (+), *n* = 69	VTE (−), *n* = 426	*p*
Age, mean (SD)	32.9 (9.4)	32.3 (8.7)	0.871
BSA, mean (SD)	2 (0.2)	2 (0.2)	0.483
BMI, mean (SD)	25.3 (4.2)	25.7 (4.5)	0.664
VTE history, *n* (%)	2 (2.9)	1 (0.2)	0.008
Smoking history, *n* (%)	24 (58.5)	136 (50.9)	0.364
Vascular compression, *n* (%)	41 (80.4)	99 (31.1)	<0.001
CTH prior to VTE, *n* (%)	29 (42.0)	426 (100.0)	<0.001
LMWH prior to VTE, *n* (%)	15 (21.7)	158 (37.1)	0.013
G‐CSF prior to VTE, *n* (%)	15 (21.7)	192 (45.1)	<0.001
CVAD, *n* (%)	3 (4.4)	5 (1.2)	0.052
ECOG PS, *n* (%)			
0–2	55 (79.7)	408 (95.8)	<0.001
3–4	14 (20.3)	18 (4.2)	
KRS, *n* (%)			
≤2	51 (73.9)	385 (90.4)	<0.001
>2	18 (26.1)	41 (9.6)	
PPS, *n* (%)			
3	11 (15.9)	193 (45.3)	<0.001
4–5	30 (43.5)	172 (40.4)	
>5	28 (40.6)	61 (14.3)	
Prior treatment, *n* (%)			
Orchiectomy	44 (63.8)	378 (88.7)	<0.001
Other surgery	25 (36.2)	74 (17.4)	<0.001
Adjuvant CTH	1 (1.5)	7 (1.6)	0.906
Adjuvant RTH	0 (0)	3 (0.7)	0.484
Histology, *n* (%)			
Seminoma	26 (37.7)	223 (52.4)	0.024
Embryonal carcinoma	31 (44.9)	235 (55.2)	0.114
Choriocarcinoma	9 (13)	48 (11.3)	0.668
Yolk sac tumour	28 (40.6)	122 (28.6)	0.045
Teratoma	20 (29)	122 (28.6)	0.953
Burned‐out tumour	5 (7.3)	11 (2.6)	0.042
Primary tumour, *n* (%)			
Testicular	58 (84.1)	395 (92.7)	0.017
Retroperitoneal	7 (10.1)	18 (4.2)	0.037
Mediastinal	3 (4.4)	8 (1.9)	0.197
Cerebral	1 (1.5)	5 (1.2)	0.846
Metastases, *n* (%)			
Nodal	30 (44.8)	271 (71.3)	<0.001
Extranodal	37 (55.2)	109 (28.7)	
CS, *n* (%)			
IS–IIIA	16 (23.2)	296 (69.5)	<0.001
IIIB–IIIC	53 (76.8)	130 (30.5)	
IGCCCG risk group, *n* (%)			
Good	21 (30.4)	324 (76.1)	<0.001
Intermediate	18 (26.1)	47 (11.0)	
Poor	30 (43.5)	55 (12.9)	
Biggest lesion, mean (SD)			
Retroperitoneal (mm)	85 (66.8)	44.4 (48.6)	<0.001
Mediastinal (mm)	25.3 (40.8)	8.1 (26.0)	

Abbreviations: BMI, body mass index; BSA, body surface area; CS, clinical stage; CTH, chemotherapy; CVAD, central venous access device; ECOG PS, Eastern Cooperative Oncology Group performance status; G‐CSF, granulocyte‐colony stimulating factor; IGCCCG, International Germ Cell Cancer Collaborative Group; KRS, Khorana risk score; LMWH, low molecular weight heparin; *n*, number; PPS, Padua Prediction Score; RTH, radiotherapy; SD, standard deviation; VTE, venous thromboembolism.

**TABLE 2:** Laboratory parameters (percentages of subgroups with complete data).

Parameter, mean (SD)	VTE (+), *n* = 69	VTE (−), *n* = 426	*p*
Hgb (g/dL)	12.6 (2.1)	14.5 (1.8)	<0.001
Plt (×10^3^/μL)	355.2 (122.1)	272.6 (97.4)	<0.001
MPV (fL)	10.1 (0.8)	10.5 (1)	<0.001
WBC (×10^3^/μL)	10.5 (4.8)	7.8 (3.1)	<0.001
Neutr (×10^3^/μL)	8.0 (4.8)	5.3 (2.9)	<0.001
Lym (×10^3^/μL)	1.4 (0.6)	1.6 (0.6)	0.016
Plt/Lym	316.1 (273)	195.6 (129.5)	<0.001
Neutr/Lym	7.5 (7.6)	3.9 (3.6)	<0.001
Mono (×10^3^/μL)	0.8 (0.3)	0.7 (0.3)	<0.001
D‐dimer (ng/mL)	5112.1 (7137.5)	1310.9 (3966.4)	<0.001
aPTT (s)	31.4 (5.5)	30 (4.4)	0.068
fibrinogen (g/L)	5.2 (2)	3.7 (1.5)	<0.001
AFP (IU/mL)	2665 (6761.9)	848.8 (4231.9)	<0.001
hCG (mIU/mL)	26804.5 (128428.4)	16023.7 (99812.1)	0.002
LDH (IU/L)	1525.3 (2676.8)	459.1 (800.7)	<0.001
GFR (mL/min/1.73 m^2^)	96.8 (34)	102 (24.4)	0.258

Abbreviations: AFP, alpha‐foetoprotein; aPTT, activated partial thromboplastin time; GFR, glomerular filtration rate; hCG, human chorionic gonadotropin; Hgb, haemoglobin; LDH, lactate dehydrogenase; Lym, lymphocytes; Mono, monocytes; MPV, mean platelet volume; Neutr, neutrocytes; Plt, platelet count; VTE, venous thromboembolism; WBC, white blood count.

### 4.2 Venous thromboembolism incidence and clinical presentation

Venous thromboembolism was diagnosed in 69 patients (13.9%), of whom DVT alone occurred in 39 (56.5%), PE alone in 16 (23.2%). Both DVT and PE was found in 14 (20.3%); in 8 cases (57.1%) synchronically and in 6 (42.9%)—metachronically. In 20 patients (29.0% of the VTE group), DVT was seen in more than one vein. Most DVT cases involved inferior vena cava (22; 31% of the whole VTE group); renal and common iliac veins accounted for 9 cases (13.0%) each and external iliac—for 8 cases (11.6%). Five and less DVT cases occurred in the following veins: femoral, popliteal, splenic, internal jugular, brachiocephalic, subclavian and internal iliac. One patient (1.4%) experienced thrombosis of a vascular access device. No patient died due to VTE.

In 40 of 69 patients (58.8%), VTE was diagnosed prior to chemotherapy and in 29 patients (42.0%) after the start of chemotherapy. In the latter, median time from Day 1 to VTE event was 49 (1–147) days.

Of 322 patients not receiving extended LMWH prophylaxis, 54 (16.8%) experienced VTE. Notably, this subgroup comprised 40 patients with VTE on presentation (hence, without prior prophylaxis) and 14 patients who commenced chemotherapy and had VTE during the treatment. Of 173 patients with extended thromboprophylaxis during chemotherapy, VTE was diagnosed in 15 (8.7%).

### 4.3 Venous thromboembolism risk factors

The following risk factors were identified in univariate analyses (Tables 3 and 4):

- Positively correlated: VTE history, ECOG PS 3‐4, surgery or trauma within 1 month prior to chemotherapy, respiratory and cardiac insufficiency, infection or rheumatological disease (the last three being PPS items), KRS ≥ 3, PPS ≥ 4, other surgery than orchiectomy prior to chemotherapy, yolk sac tumour histology, retroperitoneal primary, extranodal metastases, CS IIIB–IIIC, intermediate or poor risk according to IGCCCG, maximum dimension in the retroperitoneal space or in the mediastinum, vessels compression, Hgb decrease, Plt, WBC, Neutr and Mono, PLR, NLR, D‐dimer, fibrinogen, AFP, aPTT and LDH;
- Negatively correlated: orchiectomy, seminoma histology, testicular primary, extended LMWH prophylaxis, G‐CSF, MPV and Lym.

**TABLE 3:** Univariate analyses – patients characteristics.

Variable	OR	95% CI	*p*
Age, mean (SD)	1.01	0.98–1.04	0.617
BSA, mean (SD)	0.68	0.21–2.20	0.522
BMI, mean (SD)	0.98	0.93–1.04	0.522
VTE history, *n* (%)	12.69	1.13–141.85	0.039
Smoking history, *n* (%)	1.36	0.70–2.65	0.366
Vascular compression, *n* (%)	9.07	4.37–18.84	<0.001
CTH prior to VTE, *n* (%)	1.00		
LMWH prior to VTE, *n* (%)	0.47	0.26–0.86	0.015
G‐CSF prior to VTE, *n* (%)	0.34	0.19–0.62	<0.001
CVAD, *n* (%)	3.83	0.89–16.39	0.071
ECOG PS, *n* (%)			
0–2	1.00		
3–4	5.77	2.72–12.25	<0.001
KRS, *n* (%)			
≤2	1.00		
>2	3.31	1.77–6.20	<0.001
PPS, *n* (%)			
3	1.00		
4–5	3.06	1.49–6.29	0.002
>5	8.05	3.79–17.13	<0.001
Prior treatment, *n* (%)			
Orchiectomy	0.22	0.13–0.40	<0.001
Other surgery	2.70	1.56–4.69	<0.001
Adjuvant CTH	0.88	0.11–7.27	0.906
Adjuvant RTH	1.00		
Histology			
Seminoma	0.55	0.33–0.93	0.025
Embryonal carcinoma	0.66	0.40–1.11	0.115
Choriocarcinoma	1.18	0.55–2.53	0.668
Yolk sac tumour	1.70	1.01–2.88	0.047
Teratoma	1.02	0.58–1.78	0.953
Burned‐out tumour	2.95	0.99–8.76	0.052
Primary tumour			
Testicular	0.41	0.20–0.87	0.020
Retroperitoneal	2.56	1.03–6.38	0.044
Mediastinal	2.38	0.61–9.18	0.210
Cerebral	1.24	0.14–10.76	0.846
Metastases			
Nodal	1.00		
Extranodal	3.07	1.80–5.21	<0.001
CS			
IS–IIIA	1.00		
IIIB–IIIC	7.54	4.16–13.69	<0.001
IGCCCG risk group			
Good	1.00		
Intermediate	5.91	2.93–11.90	<0.001
Poor	8.42	4.50–15.75	<0.001
Biggest lesion mean (SD)			
Retroperitoneal (mm)	7.54	4.16–13.69	<0.001
Mediastinal (mm)	1.01	1.01–1.02	<0.001

Abbreviations: BMI, body mass index; BSA, body surface area; CI, confidence interval; CS, clinical stage; CTH, chemotherapy; CVAD, central venous access device; ECOG PS, Eastern Cooperative Oncology Group performance status; G‐CSF, granulocyte‐colony stimulating factor; IGCCCG, International Germ Cell Cancer Collaborative Group; KRS, Khorana risk score; LMWH, low molecular weight heparin; *n*, number; OR, odds ratio; PPS, Padua Prediction Score; RTH, radiotherapy; SD, standard deviation.

**TABLE 4:** Univariate analyses – laboratory parameters.

Variable	OR	95% CI	*p*
Hgb (per 1 g/dL decrease)	1.52	1.35–1.72	<0.001
Plt (×10^3^/μL)	1.01	1.00–1.01	<0.001
MPV (fL)	0.59	0.43–0.81	0.001
WBC (×10^3^/μL)	1.18	1.11–1.26	<0.001
Neutr (×10^3^/μL)	1.20	1.12–1.28	<0.001
Lym (×10^3^/μL)	0.55	0.34–0.87	0.011
Plt/Lym	1.00	1.00–1.01	<0.001
Neutr/Lym	1.13	1.08–1.19	<0.001
Mono (×10^3^/μL)	3.36	1.63–6.91	0.001
D‐dimer (ng/mL)	1.00	1.00–1.00	<0.001
aPTT (s)	1.06	1.00–1.12	0.034
Fibrinogen (g/L)	1.58	1.34–1.86	<0.001
AFP (IU/mL)	1.00	1.00–1.00	0.011
hCG (mIU/mL)	1.00	1.00–1.00	0.435
LDH (IU/L)	1.00	1.00–1.00	<0.001
GFR (mL/min/1.73 m^2^)	0.99	0.98–1.00	0.119

Abbreviations: AFP, alpha‐foetoprotein; aPTT, activated partial thromboplastin time; CI, confidence interval; GFR, glomerular filtration rate; hCG, human chorionic gonadotropin; Hgb, haemoglobin; LDH, lactate dehydrogenase; Lym, lymphocytes; Mono, monocytes; MPV, mean platelet volume; Neutr, neutrocytes; OR, odds ratio; Plt, platelet count; WBC, white blood count.

### 4.4 KRS and PPS

According to KRS, 11.9% patients had high VTE risk (KRS ≥ 3) and the remaining 88.1% had intermediate risk. VTE incidence was 30.5% (18 out of 59) and 11.7% (51 out of 436) in the high‐risk and intermediate‐risk group, respectively. Of all VTE events, 26.1% (18 out of 69) occurred in high‐risk patients.

According to PPS, 18.0%, 40.8% and 41.2% patients scored >5, 4–5 and 3, respectively. VTE incidence in the subgroups was 31.5% (28 out of 89), 14.9% (30 out of 202) and 5.4% (11 out of 204). Of all VTE events, 40.6% (28 out of 69) and 43.5% (30 out of 69) occurred in patients scoring >5 and 4–5, respectively. If the original PPS cut‐off value (≥4 and <4) were applied VTE incidence in the high‐risk group would be 19.9% (58 out of 291) and in the low‐risk group – 5.4% (11 out of 204). In this case, 84.1% of all VTE events (58 out of 69) would occur in high‐risk (≥4) patients.

Both KRS and PPS proved useful in VTE prediction in GCT patients. KRS ≥ 3 significantly correlated with increased VTE risk (OR: 3.31; 95% CI: 1.77–6.20; *p* < 0.001). Similar correlations were obtained for PPS 4–5 (OR: 3.06; 95% CI: 1.49–6.29; *p* = 0.002) and PPS > 5 (OR: 8.05; 95% CI: 3.79–17.13; *p* < 0.001).

AUC‐ROC statistics favoured PPS over KRS: 0.700 (acceptable) versus 0.588 (low). AIC and BIC for PPS were 0.799 and (−1585), whereas for KRS – 0.839 and (−1576), which means that, again, PPS fared better.

### 4.5 Multivariate models

Six models were constructed and subjected to multivariate analysis (Table 5). The following variables were grouped into seven‐item sets in various configurations: ECOG PS, seminoma and yolk sac tumour histology, CS IIIB–IIIC, IGCCCG intermediate or poor risk group, extranodal metastases, vascular compression, Hgb, Plt, MPV and Mono. Each model was adjusted for LMWH prophylaxis. The models were then subjected to forward stepwise regression (Table 6). The best fitting models were:

- ‘complete’ Model 2 (ECOG PS, seminoma, yolk sac tumour, CS, vessels compression, Hgb, LMWH prophylaxis)
- ‘selected’ Models 1, 5, 6 (Hgb, IGCCCG risk group).

**TABLE 5:** Multivariate models (‘complete’).

No.	Variables	OR	95% CI	*p*	AUC	AIC	BIC
1	ECOG PS	0.41	0.11–1.55	0.188	0.878	0.572	−1639
	Seminoma	0.91	0.37–2.25	0.838			
	Yolk sac tumour	0.75	0.30–1.89	0.542			
	Prognostic group	4.31	1.50–12.36	0.007			
	Vein compression	11.28	3.72–34.20	<0.001			
	Hgb	1.33	1.06–1.67	0.014			
	LMWH prophylaxis	0.05	0.02–0.14	<0.001			
2	ECOG PS	0.43	0.11–1.63	0.212	0.885	0.567	−1641
	Seminoma	0.70	0.29–1.67	0.422			
	Yolk sac tumour	0.79	0.31–2.01	0.620			
	CS	5.68	1.82–17.70	0.003			
	Vein compression	8.96	2.85–28.13	<0.001			
	Hgb	1.32	1.03–1.67	0.026			
	LMWH prophylaxis	0.04	0.02–0.12	<0.001			
3	ECOG PS	0.52	0.16–1.75	0.294	0.875	0.617	−1624
	Yolk sac tumour	1.15	0.49–2.67	0.749			
	CS	12.61	4.46–35.66	<0.001			
	Hgb	1.49	1.19–1.89	<0.001			
	MPV	1.00	0.65–1.54	0.996			
	Monocytes	0.68	0.17–2.65	0.577			
	LMWH prophylaxis	0.08	0.03–0.21	<0.001			
4	ECOG PS	0.45	0.12–1.69	0.234	0.885	0.567	−1641
	Yolk sac tumour	0.84	0.34–2.06	0.705			
	CS	5.64	1.84–17.33	0.002			
	Vein compression	9.37	3.02–29.07	<0.001			
	Hgb	1.27	0.99–1.61	0.064			
	Plt	1.00	1.00–1.01	0.440			
	LMWH prophylaxis	0.04	0.01–0.12	<0.001			
5	Yolk sac tumour	0.88	0.38–2.02	0.756	0.862	0.635	−1619
	Prognostic group	8.35	3.05–22.86	<0.001			
	Extranodular mets	0.68	0.27–1.68	0.398			
	Hgb	1.47	1.19–1.82	<0.001			
	Plt	1.00	1.00–1.01	0.396			
	MPV	1.07	0.68–1.69	0.763			
	LMWH prophylaxis	0.10	0.04–0.27	<0.001			
6	Seminoma	0.93	0.37–2.29	0.868	0.872	0.572	−1639
	Yolk sac tumour	0.82	0.32–2.07	0.672			
	Prognostic group	4.63	1.59–13.49	0.005			
	Vein compression	12.23	3.96–37.73	<0.001			
	Hgb	1.30	1.04–1.61	0.020			
	Monocytes	0.41	0.10–1.72	0.221			
	LMWH prophylaxis	0.05	0.02–0.15	<0.001			

Abbreviations: AIC, Akaike information criterion; AUC, area under curve; BIC, Bayesian information criterion; CI, confidence interval; CS, clinical stage; ECOG PS ‐ Eastern Cooperative Oncology Group performance status; Hgb, haemoglobin; LMWH, low molecular weight heparin; mets, metastases; MPV, mean platelet volume; No., number; OR, odds ratio; Plt, platelet count.

**TABLE 6:** Multivariate models (‘selected’).

No.	Variables	OR	95% CI	*p*	AUC	AIC	BIC
1, 5, 6	Hgb	1.41	1.18–1.67	<0.001	0.830	0.693	−1619
	Prognostic group	3.90	1.74–8.73	0.001			
2	CS	9.46	4.37–20.47	<0.001	0.767	0.728	−1608
	Seminoma	0.68	0.35–1.33	0.258			
3, 4	CS	9.95	4.62–21.43	<0.001	0.754	0.726	−1612

Abbreviations: AIC, Akaike information criterion; AUC, area under curve; BIC, Bayesian information criterion; CI, confidence interval; CS, clinical stage; Hgb, haemoglobin; No., number; OR, odds ratio.

**FIGURE 1:**
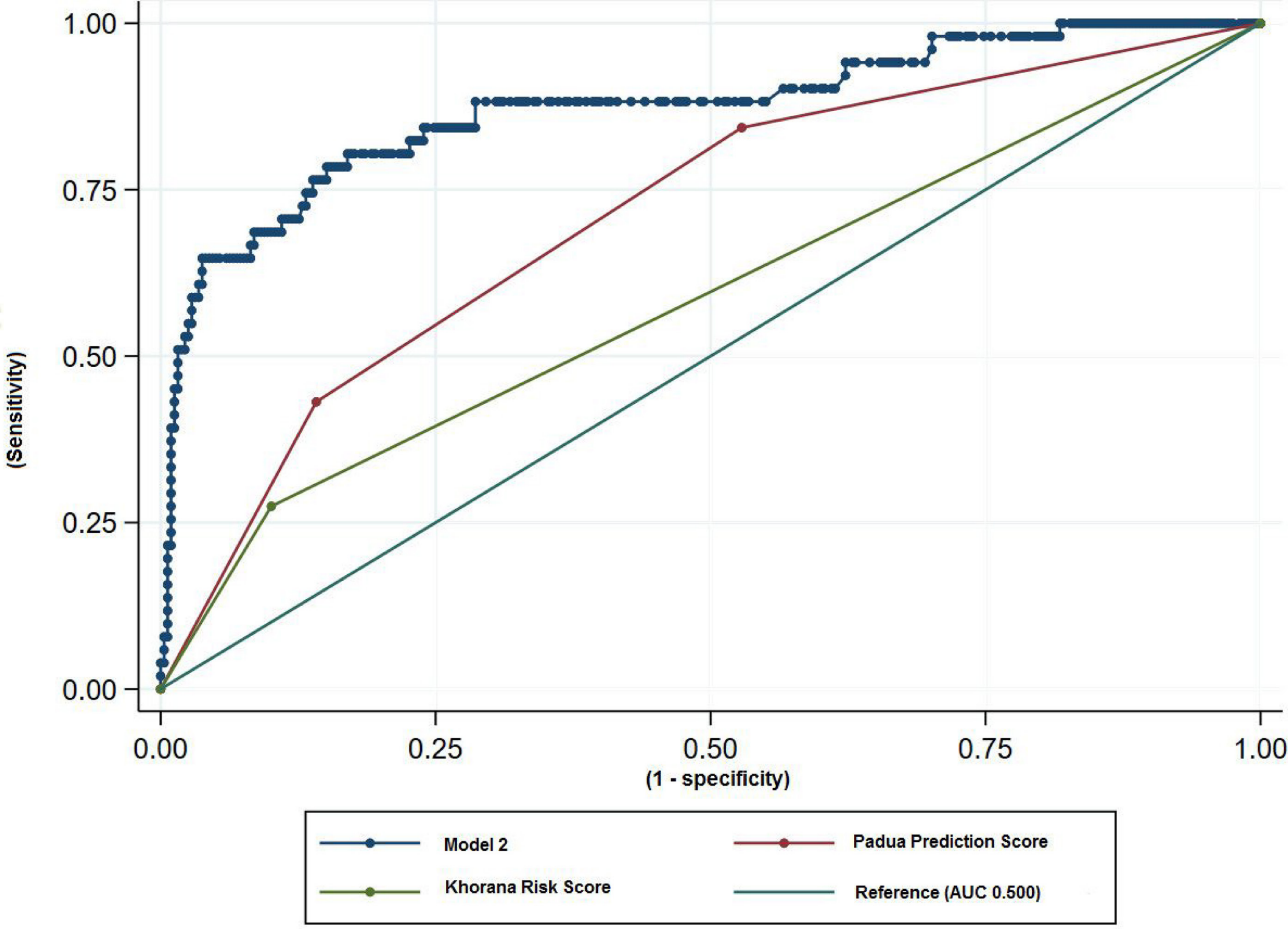
Receiver‐operating characteristic (ROC) curves for the developed risk assessment model (RAM), Khorana risk score (KRS) and Padua Prediction Score (PPS). AUC, area under curve.

The ‘complete’ model, with AUC‐ROC 0.885, AIC 0.567 and BIC (−1641), fared better than the ‘selected’ model. Both, however, yielded better results in terms of VTE risk prediction than either KRS or PPS (Figure 1). Moreover, overfitting statistics also favoured tested models.

### 4.6 LMWH prophylaxis

In all six models, LMWH prophylaxis significantly reduced VTE risk. The magnitude of LMWH effect was similar in all the models; in Model 2, OR for VTE risk was 0.04 (95% CI: 0.02–0.12; *p* = 0.001).

### 4.7 Risk assessment score

All significant variables from Model 2, excluding LMWH prophylaxis, were used to develop a numerical risk score. As planned, it is on the basis of the risk score that the decision concerning thromboprophylaxis will be made. The three items in Model 2 had the following ORs:

- vessels compression: OR: 8.96 (95% CI: 2.85–28.13; *p* < 0.001).
- CS IIIB–IIIC: OR: 5.68 (95% CI: 1.82–17.70; *p* = 0.003).
- Hgb: OR: 1.32 (95% CI: 1.03–1.67).

**FIGURE 2:**
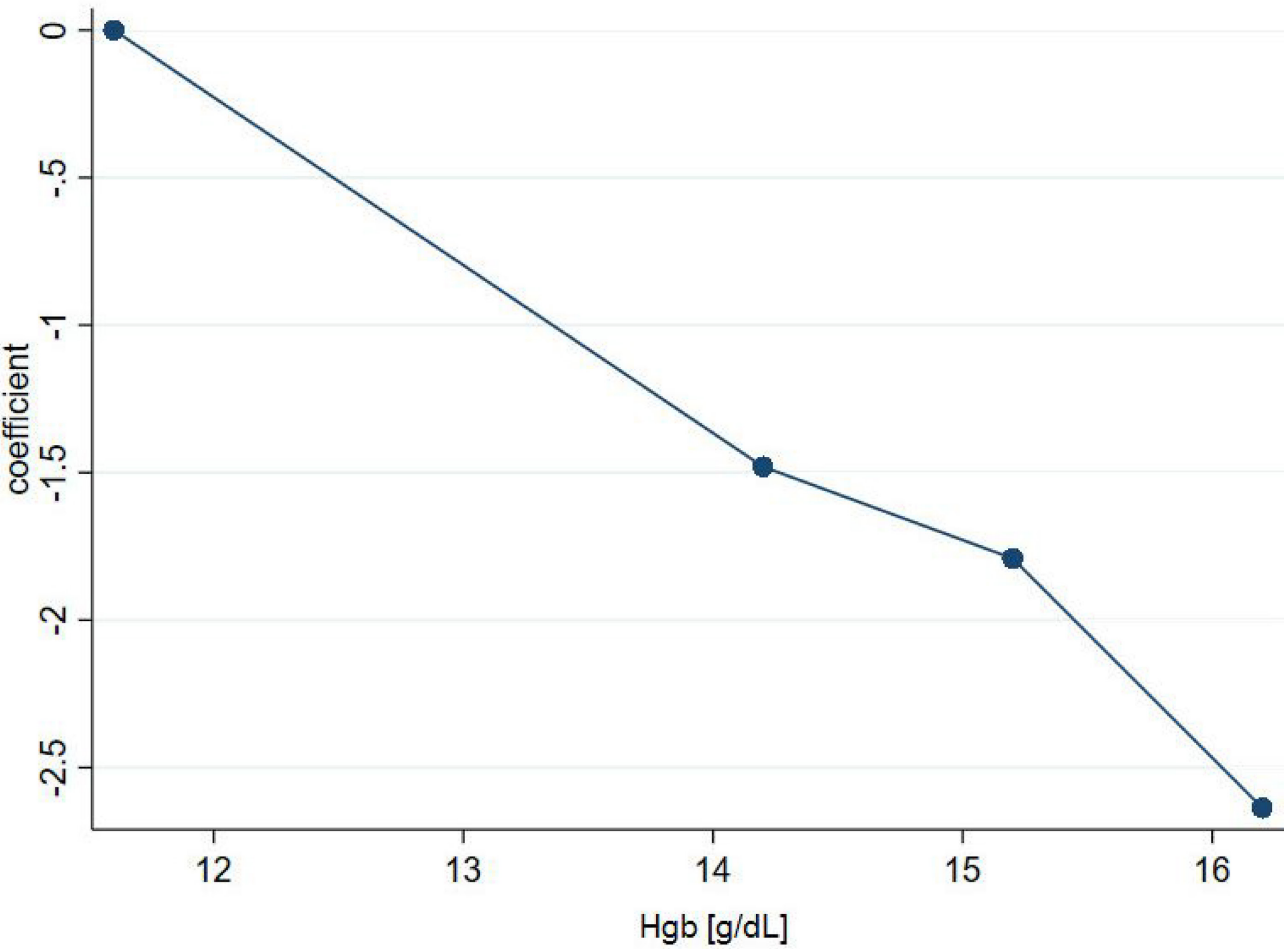
Regression coefficient for haemoglobin concentration. g/dL, grams per decilitre.

**FIGURE 3:**
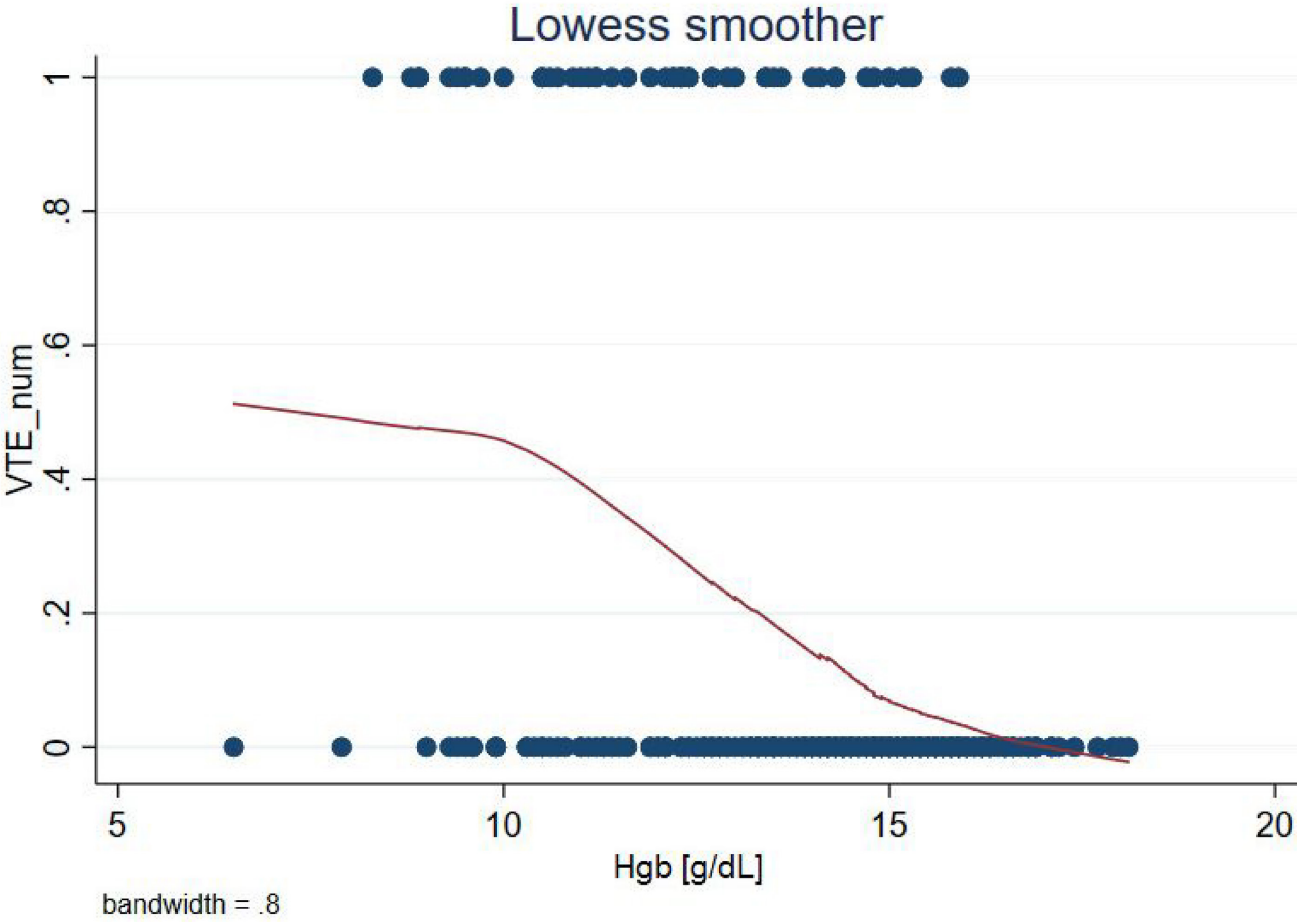
Locally weighted scatterplot smoothing (LOWESS). g/dL, grams per decilitre; num, number; VTE, venous thromboembolism.

According to the logistic regression coefficient as well as LOWESS analysis, the correlation between Hgb and VTE risk was nearly linear (Figures 2 and 3). In other words, OR only depended on the difference between any two Hgb concentrations and not on their absolute values. Hence, even within reference laboratory values for Hgb, the associated VTE risk may differ.

We did not find any cut‐off value for Hgb that would improve the model's statistics. All estimated models with binary Hgb showed lower AUC‐ROC and higher deviance (Table 7). Therefore, Hgb was left as a continuous variable. The risk score was then constructed on the basis of ORs and regression coefficients of all three variables (Table 8).

**TABLE 7:** c‐statistics and deviance for Hgb as a continuous or binary variable.

Hgb – type of variable	AUC‐ROC	Deviance
Continuous	0.8851	165.88
Binary (cut‐off value; g/dL)		
10.0	0.8751	170.54
11.0	0.8761	169.74
11.5	0.8759	169.72
12.0	0.8744	169.95

Abbreviations: AUC‐ROC, area under receiver operating characteristic curve; Hgb, haemoglobin.

**TABLE 8:** VTE risk assessment score for metastatic GCT patients.

Risk factor	Score
Vascular compression (CT)	7
Clinical stage IIIB–IIIC	4
Haemoglobin concentration only if below the lower limit of normal: the difference between lower limit of normal and patient's concentration (LLN – [Hgb]), rounded up to integer	1 for every 1 g/dL

Abbreviations: CT, computed tomography; GCT, germ cell tumour; Hgb, haemoglobin; LLN, lower limit of normal; VTE, venous thromboembolism.

In 369 patients with complete data regarding vein compression, scores ranged from 0 to 19. In total, 190 patients (51%) scored 0; VTE incidence in this subgroup was 2.6%. Cut‐off point of ≥1 generated VTE risk of 26%; scores ≥2, ≥3, and ≥4–28%; ≥5, ≥6, and ≥7–29%; ≥8–34%, ≥9–35%, ≥10 and ≥11–36%, ≥12–38% and ≥13–41%. Sensitivity and specificity ranges for consecutive scores were 0.90–0 and 0.58–1.00, respectively. The optimal cut‐off point for high‐risk group, with minimal absolute difference (and maximum product) between sensitivity (0.78) and specificity (0.77) was ≥9, with positive and negative predictive values of 0.35 and 0.96, respectively. VTE incidence in low‐ and high‐risk patients (i.e. <9 and ≥9) was 4.3% and 35.4%, respectively.

## 5 DISCUSSION

VTE incidence in our cohort was 13.9%; in other GCT groups ranged from 3% to 28%.^14^, ^15^, ^16^, ^17^, ^18^, ^19^, ^20^, ^21^, ^22^, ^23^, ^24^, ^25^, ^26^, ^27^, ^28^, ^29^, ^30^, ^31^ Differences resulted from patients' selection criteria, including or excluding cases with VTE prior to chemotherapy or with LMWH prophylaxis.

Interestingly, 58% VTE cases in our group were diagnosed prior to chemotherapy. Other authors usually reported contrary results; pre‐treatment VTE accounted for 13.0%–44.7% of all events.^15^, ^18^, ^21^, ^22^, ^23^, ^25^, ^30^ In the group of Honecker et al,^14^ 81% of VTE cases occurred before first‐ or second‐line chemotherapy. However, if first‐line patients were considered separately, pre‐chemotherapy VTE accounted for 54.5%, which is consistent with our paper. Patients' characteristics in the group of Tran et al. matched ours in many aspects, including VTE incidence – 13%.^15^ Thromboprophylaxis was divided into short‐ and long‐term (<7 and ≥7 days, respectively) but patients receiving long‐term prophylaxis (7%) were then excluded. Follow‐up spanned 90 days,^15^ half the period that we adopted; still, only 5.8% VTE events occurred in our patients after 90 days.

VTE risk does not remain constant in the course of disease. According to Lauritsen et al,^20^ hazard ratio (HR) for VTE was 24.7 (95% CI: 14.0–43.6) during the first year following BEP chemotherapy and decreased to 1.4 (95% CI: 0.9–2.2) after 10 years.^20^ We deliberately analysed pre‐chemotherapy values only, as the goal of our study was to construct a RAM that would guide an upfront decision on thromboprophylaxis in new GCT patients. Still, some on‐treatment circumstances may trigger VTE, for example, hospitalisation, sepsis or immobilisation. Periodic re‐assessment of VTE risk and indications for thromboprophylaxis is therefore advised.

GCT histological entities have rarely been investigated separately as potential VTE risk factors. In our study, seminoma histology surprisingly decreased VTE risk whereas yolk sac tumour showed a contrary correlation. Other types proved insignificant. However, neither seminoma nor yolk sac tumour retained significance in multivariate analyses. Detailed histology was also investigated by Honecker et al^14^: seminoma correlated with a higher VTE risk but only in univariate analysis.

Most authors restricted themselves to seminomas and non‐seminomas. According to Bezan et al,^19^ non‐seminomatous histology significantly increased VTE risk in univariate (HR: 4.23; 95% CI: 1.88–9.49; *p* < 0.0001) but not in multivariate analysis. Importantly, the analysis of CS IS–IIIC subgroup did not reveal any correlation from the start.^19^ Gizzi et al. did not show a correlation of non‐seminoma histology with VTE risk (OR: 1.39; 95% CI: 0.43–4.56; *p* = 0.58).^28^ On the other hand, seminoma histology increased VTE risk in the groups of Robinson et al^22^ (OR: 2.14; 95% CI: 1.11–4.14; *p* = 0.023) and Piketty et al^27^ (OR: 3.4; *p* = 0.01), but significance was lost in multivariate analyses.^22^, ^27^ Moreover, only 35% in the former group received chemotherapy.^22^

As expected, CS significantly correlated with a higher VTE risk, most probably due to the tumour volume, concentration of serum coagulants and vessels compression (analysed here as a separate factor). We assumed both CS IIIB–IIIC (in reference to CS IS–IIIA) and extranodal metastases to be potential VTE risk factors, which proved correct in univariate analyses. CS was then investigated in three of six RAMs and proved significant. Additionally, it retained its significance after the stepwise forward selection procedure, with a stronger correlation than in the available literature. In contrast, extranodal metastases, analysed in one model, lost their effect.

Our findings are in line with other studies. A higher VTE risk was correlated with CS III,^30^ CS > I (OR: 16.95; 95% CI: 3.49–82.38; *p* < 0.001)^32^ or CS ≥IIC (OR: 2.259; 95% CI: 1.105–4.618; *p* = 0.026).^16^ Moreover, comparable results were obtained for parenchymal extrapulmonary metastases (OR: 3.297; 95% CI: 1.353–5.077; *p* = 0.025) and regional lymph nodes ≥N2 (OR: 3.478; 95% CI: 1.126–10.745; *p* = 0.030).^16^ Bezan et al^19^ investigated VTE risk in TGCT patients staged IS–IIC and IIIA–IIIC in reference to CS IA–IB. Both ranges of higher CS correlated with a higher VTE risk in univariate analysis, but this significance was only retained for CS IIIA–IIIC in multivariate analysis (HR: 4.87; 95% CI: 1.97–12.00; *p* = 0.001).^19^ Honecker et al^14^ found that metastases in retroperitoneal and supraclavicular lymph nodes correlated with VTE risk, contrarily to mediastinal, pulmonary and hepatic metastases. Only supraclavicular localisation remained significant in multivariate analysis (*p* = 0.03).^14^ Of note, some authors found CS insignificant for VTE risk.^26^, ^27^, ^28^

Intermediate and poor risk IGCCCG prognostic groups correlated with a higher VTE risk in univariate analyses; three of six models comprised this variable. Together with Hgb, prognostic group retained its significance in the forward step regression analysis. To the best of our knowledge, ours is the second study in which prognostic group yielded such results; most studies investigated TNM CS. These two classifications systems are overlapping to some extent. However, due to dissimilarities, we analysed CS and IGCCCG groups separately. Corresponding results were obtained by Bezan et al^19^; intermediate or poor risk patients presented with a higher VTE risk (OR: 2.61; 95% CI: 1.16–5.88; *p* = 0.02), confirmed in a validation group.^19^ Srikanthan et al.^18^ only subjected the prognostic group to univariate analysis. Intermediate or poor risk group significantly increased VTE risk (OR: 3.76; 95% CI: 1.50–9.46; *p* = 0.005) but not in a validation cohort.^18^ Similarly, Tran et al^15^ observed an increase in VTE risk for intermediate (OR: 1.72; 95% CI: 1.08–2.74; *p* = 0.02) and poor risk group (OR: 3.26; 95% CI: 2.06–5.17; *p* < 0.001) but only in univariate analyses.^15^ Intermediate but not poor risk group increased VTE risk in the study of Honecker et al^14^; this finding was not confirmed in multivariate analysis. The results may have been affected by including adjuvant chemotherapy and recurring cases (48% of the study population).^14^ Some authors failed to find a significant correlation.^16^, ^17^, ^21^, ^26^, ^27^, ^28^

Deceleration of blood flow, usually due to vessels compression, has been a renowned VTE risk factors since Virchow's triad.^7^, ^33^ In our univariate analyses, vessels compression yielded the highest OR (9.07; 95% CI: 4.37–18.84; *p* < 0.001) and was further investigated in four of six multivariate models. The significance was confirmed with even higher ORs: 11.28; 8.96; 9.37 and 12.23. However, analyses of this variable have limitations. Retrospectively, we were only able to analyse CT reports, not images. Many patients referred to our department had their CT scans performed elsewhere. High ORs obtained in the multivariate models indicated that vessels compression was independent of GCT‐specific risk factors. We hence postulate that it must be stated directly in CT reports whether vessels compression is present or not.

LMWH prophylaxis in our study was administered in 21.7% patients in the VTE (+) group and in 37.1% in the VTE (−) group. In univariate analysis, LMWH reduced VTE risk approximately two‐fold. In all multivariate models, this effect remained significant; in the best‐fitting model (Model 2) OR was 0.04 (95% CI: 0.02–0.12; *p* < 0.001). However, administration of LMWH was left to the decision of the attending physician, based on renowned risk factors and RAMs, usually KRS. LMWH prophylaxis was not based on uniform criteria, medications and doses differed and intermittent administration was not infrequent. Therefore, our results on LMWH influence should be treated with caution.

Bezan et al^19^ estimated number needed to treat (NNT) for GCT patients on prophylaxis for CS IA–IB, IS–IIB, IIC and IIIA–IIIC: 118, 34, 14 and 9, respectively. Number needed to harm (NNH) was 125.^19^ Fankhauser et al^23^ obtained similar results; the benefit was even more pronounced in patients with central venous access.^23^ Both trials corroborate safety and efficacy of thromboprophylaxis.

On the other hand, many authors failed to reproduce these results. In GCT patients receiving in‐hospital or extended thromboprophylaxis, Solari et al. found no correlation between LMWH administration and VTE incidence.^29^ In the studies of Gizzi et al^28^ and Haugnes et al^21^ thromboprophylaxis had no effect on VTE; moreover, bleeding risk was significantly increased in the LMWH group (14.5% vs. 1.1%; *p* < 0.001).^21^ In other studies, thromboprophylaxis was administered in 4%^34^ to 93%^32^ patients but did not affect VTE risk.^14^, ^16^, ^27^, ^34^, ^35^, ^36^, ^37^

Anaemia has been a renowned VTE risk factor and one of the KRS items.^9^, ^38^ In our cohort, mean Hgb was significantly lower (by 1.9 g/dL) in VTE subgroup. It was then analysed as a continuous variable in all six multivariate models and proved significant in five. In comparison with vessel compression and CS, anaemia was the weakest risk factor.

Other researchers usually analysed Hgb as a binary variable, with different cut‐off points: <13 g/dL (OR for VTE: 4.20; 95% CI: 1.48–14.51; *p* = 0.012)^39^ or <10 g/dL (OR: 1.78; 95% CI: 1.28–2.47).^40^ Moore et al. found that Hgb < 10 g/dL or treatment with erythropoietins correlated with VTE risk in univariate (*p* = 0.03) but not in multivariate analysis.^34^ In two GCT cohorts^18^, ^19^ Hgb proved insignificant for VTE risk.

All patients in our study scored one point in KRS due to cancer type; hence, there were no low‐risk patients. KRS ≥ 3 correlated with a higher VTE risk but AUC‐ROC for KRS was inferior to our model (0.558 vs. 0.885). This might result from strong correlations of vessel compression and CS. Of all VTE (+) patients in our cohort, 26.1% (18 out of 69) had a high‐risk KRS (≥3); the majority of VTE cases were diagnosed in intermediate‐risk patients. This corresponds with meta‐analysis of Mulder et al,^41^ where 23.4% VTE (+) patients presented with a high‐risk KRS. The authors concluded that KRS sufficiently identified high risk patients, especially within 6 months, but the prediction in the intermediate risk group was suboptimal.^41^ In our study, VTE incidence in the intermediate risk group was 11.7% (51/436), which is in line with meta‐analysis of Bao et al – 11%.^42^ These authors reported on fewer VTE cases among KRS high risk (≥3) patients – 14%^42^ versus 30.5% (18 out of 59) in our group.

Several studies addressed the utility of KRS in GCT patients. KRS ≥ 3 correlated with an increased VTE risk; OR ranged from 2.62 (95% CI: 1.28–5.35; *p* = 0.0008)^15^ to 11.80 (95% CI: 3.93–35.39; *p* < 0.001).^18^ Bezan et al^19^ applied the threshold of ≥2; after correction for chemotherapy, VTE risk was on the verge of significance (HR: 2.22; 95% CI: 1.02–4.85; *p* = 0.05). Furthermore, when corrected for CS, KRS was insignificant.^19^ In the study of Heidegger et al, only 7.7% of VTE cases occurred in patients scoring ≥3,^17^ which was less than in the above‐mentioned meta‐analysis.^42^ Other authors^21^, ^43^ did not show a correlation between KRS and VTE risk in GCT patients at all.

PPS < 4 and ≥4 identify low and high VTE risk patients, respectively. All our patients scored three points for an active malignancy and some scored additional two points for surgery within the last month. In result, even patients without other VTE risk factors scored five points only due to orchiectomy, bearing a low VTE risk. We therefore decided to analyse two cut‐off levels, that is, three subgroups: PPS 3 (reference group), PPS 4–5 and >5. Both tested ranges correlated with a higher VTE risk: OR: 2.91 (95% CI: 1.22–6.92; *p* = 0.016) and 7.64 (95% CI: 3.17–18.38; *p* < 0.001), respectively. PPS proved inferior to our model (AUC‐ROC: 0.700 vs. 0.885) but superior to KRS.

In the study of Germini et al,^44^ PPS ≥ 4 significantly correlated with VTE risk (OR: 4.56; 95% CI: 2.68–7.74; *p* = 0.000) and allowed for a more accurate VTE prediction than individual assessment by attending physicians. However, there were few patients with active malignancies (3.8% and 6.9%).^44^ Zhou et al^45^ found PPS superior (AUC‐ROC: 0.716; 95% CI: 0.693–0.740) to Caprini risk score. The authors concluded that routine thromboprophylaxis in patients with PPS ≥4 (OR for VTE: 5.01; 95% CI: 4.03–6.25; *p* < 0.001) was justified. Moreover, PPS ≥4 correlated with higher mortality during hospitalisation and 6 months after (16.6%; *p* < 0.001).^45^ This effect was confirmed by Vardi et al^46^ in hospitalised patients with sepsis, of whom 16.7% had a malignancy. PPS ≥4 correlated with in‐hospital mortality (OR: 6.08; 95% CI: 3.73–9.92; *p* < 0.0001) and overall survival (OS) but not with VTE risk; correction for thromboprophylaxis did not affect these results. Moreover, VTE incidence did not correlate with mortality or OS.^46^

To the best of our knowledge, ours is the third study involving GCT patients where a RAM was developed and compared to established risk scores. Moreover, it seems to be the first in which a numerical scale was constructed. Srikanthan et al^18^ proffered retroperitoneal lymph nodes (RPLN) >5 cm as a single factor for VTE risk assessment; it proved superior to KRS (AUC‐ROC: 0.71 vs. 0.67 in the training cohort, respectively, and 0.61 vs. 0.57 in the validation cohort).^18^ In the study of Bezan et al,^19^ VTE risk increased with CS: HR 3.45 (95% CI: 1.13–10.53; *p* = 0.03) for IS–IIB; 8.86 (95% CI: 2.35–33.45; *p* = 0.001) for IIC and 13.82 (95% CI: 5.88–32.51; *p* < 0.0001) for IIIA–IIIC. CS was more precise than RPLN in VTE prediction (AUC‐ROC: 0.75 vs. 0.63; *p* = 0.007).^19^ Meng et al recommended thromboprophylaxis in GCT patients undergoing cisplatin‐based chemotherapy in case of KRS ≥ 3, RPLN ≥ 5 cm, CS ≥IIC, intermediate IGCCCG risk group or LDH ≥ 1.5× upper reference limit. On absence of the above factors, prophylaxis was advised if chemotherapy was delivered through a central line. However, this paper was a systematic analysis of eight chosen studies and did not involve an original patients' cohort.^47^

Comprising three rudimentary items, our risk score is straightforward and easy to operate. Whether its superiority over KRS and PPS can be sustained in a validation cohort remains to be seen. Our results echo other studies^18^, ^19^; VTE risk increased with CS and vessel compression (which is believed to occur more often in RPLN > 5 cm). Hgb was no surprise here; yet, strikingly, its correlation with VTE risk turned out to be approximately linear along any given concentration range. In other words, even within normal Hgb limits (14.0–18.0 g/dL) VTE risk can change considerably. Post hoc analyses with Hgb as a binary variable resulted in decreasing model's fitness. In the literature, Hgb has been usually managed categorically, with a given cut‐off point, e.g., 10 g/dL, as in KRS. Allegedly, Hgb treated as a continuous variable may be the key to better prediction of CAT.

The cut‐off point of ≥9 could suggest possible target patients for prophylaxis; however, we should keep in mind that some patients scored 7 only due to vein compression, which, in line with Virchow's triad, turned out to be the strongest predictive factor. Recommendation of prophylaxis in patients ≥7 would also be sensible. Last but not least, our results must be confirmed in a validation cohort.

The main limitation of our study is its retrospective design. There were missing data, we could only rely on CT reports instead of scans and LMWH prophylaxis was a physician's subjective decision, hence the choice of drug and dosing were not uniform. The number of VTE events limited the maximal number of variables in multivariate analyses. Hence, some variables significant in univariate analyses were not further investigated.

All data were collected from one time point, that is, before chemotherapy. Such approach was in line with the aim to be accomplished, that is, assess VTE risk using baseline parameters. On the other hand, there has been more emerging evidence that dynamic re‐assessment over the course of treatment results in better VTE prediction.^24^, ^48^, ^49^

Strengths of our study include the population's size from one centre and the number of variables analysed. Our VTE risk assessment model may have a substantial contribution to everyday practice in GCT clinic, that is, more targeted thromboprophylaxis.

## 6 CONCLUSIONS

Our RAM based on vein compression, clinical stage and haemoglobin concentration proved superior to both KRS and PPS in terms of discriminatory power and risk of overfitting. A validation cohort is currently being collected and the numerical score will be further tested. Should our outcomes be corroborated, the presented risk score will have the highest predictive value so far in GCT patients.

## ETHICS APPROVAL STATEMENT

The study conforms to the Declaration of Helsinki and was approved by the Ethical Committee at Maria Sklodowska‐Curie National Research Institute of Oncology, Warsaw, Poland (consent no. 16/2021; 25 March, 2021). Only retrospective data were analysed. All prior medical procedures, examinations of biological materials, diagnostic imaging, pathology, radiology and laboratory reports, as well as data from medical charts had constituted standard clinical management of germ cell tumour patients.

## AUTHOR CONTRIBUTIONS

**Wojciech Michalski:** Conceptualization (lead); data curation (lead); formal analysis (lead); investigation (lead); methodology (equal); project administration (lead); resources (lead); supervision (lead); writing – original draft (lead); writing – review and editing (lead). **Grażyna Poniatowska:** Conceptualization (supporting); investigation (supporting); methodology (supporting); writing – review and editing (supporting). **Joanna Jońska‐Gmyrek:** Data curation (supporting); formal analysis (supporting); investigation (supporting); writing – review and editing (supporting). **Agnieszka Żółciak‐Siwińska:** Formal analysis (supporting); methodology (supporting); writing – review and editing (supporting). **Inga Zastawna:** Formal analysis (supporting); methodology (supporting); supervision (supporting); writing – review and editing (supporting). **Artur Lemiński:** Formal analysis (supporting); writing – review and editing (supporting). **Anna Macios:** Conceptualization (supporting); formal analysis (supporting); methodology (equal); writing – review and editing (supporting). **Michał Jakubczyk:** Formal analysis (supporting); methodology (supporting); writing – review and editing (supporting). **Tomasz Demkow:** Formal analysis (supporting); writing – review and editing (supporting). **Paweł Wiechno:** Conceptualization (supporting); data curation (supporting); formal analysis (supporting); investigation (supporting); methodology (supporting); project administration (equal); supervision (lead); writing – review and editing (supporting).

## FUNDING INFORMATION

No funding was obtained to support this study.

## CONFLICT OF INTEREST STATEMENT

AL declared speaker honoraria and support for attending meetings and/or travel from Ipsen. All the remaining authors declared no potential conflict of interest associated with this publication.

## Data Availability

The data that support the findings of this study are available on request from the corresponding author. The data are not publicly available due to privacy or ethical restrictions.
